# Supplementary material for: Direct sequencing of Leishmania donovani from patients in Garissa County, Northern Kenya, reveals a newly emerging intra-specific hybrid genotype
Source: PLoS Negl Trop Dis. 2026 Jan 27;20(1):e0013144. doi: 10.1371/journal.pntd.0013144 (PMC12875589; doi:10.1371/journal.pntd.0013144)
Supplement: S1 Table — (DOCX) [file pntd.0013144.s001.docx]

| **Sample ID** | **Gender** | **Home location** | **County** | **Year of collection** | **Other information** |
| --- | --- | --- | --- | --- | --- |
| GSA 010 | Male | Saka | Garissa | 2019 | rk39 antigen +ve, pancytopenia, headache, fever, hepatosplenomegaly, jaundance |
| GSA 047 | Male | Ngomeni -mwingi | Kitui | 2019 | Pancytopenia |
| GSA 067 | Male | Ngomeni -mwingi | Kitui | 2020 | Hepatosplenomegaly |
| GSA 220 | Male | Balambala | Garissa | 2021 | Hepatosplenomegaly |
| GSA 231 | Male | Ngomeni -mwingi | Kitui | 2021 | N/A |
| GSA 248 | Male | Ngomeni -mwingi | Kitui | 2021 | N/A |
| GSA 265 | Male | Balambala | Garissa | 2022 | N/A |
| GSA 266 | Female | Ngomeni -mwingi | Kitui | 2022 | N/A |
