# Supplementary figures and images for: Direct sequencing of Leishmania donovani from patients in Garissa County, Northern Kenya, reveals a newly emerging intra-specific hybrid genotype
Source: PLoS Negl Trop Dis. 2026 Jan 27;20(1):e0013144. doi: 10.1371/journal.pntd.0013144 (PMC12875589; doi:10.1371/journal.pntd.0013144)

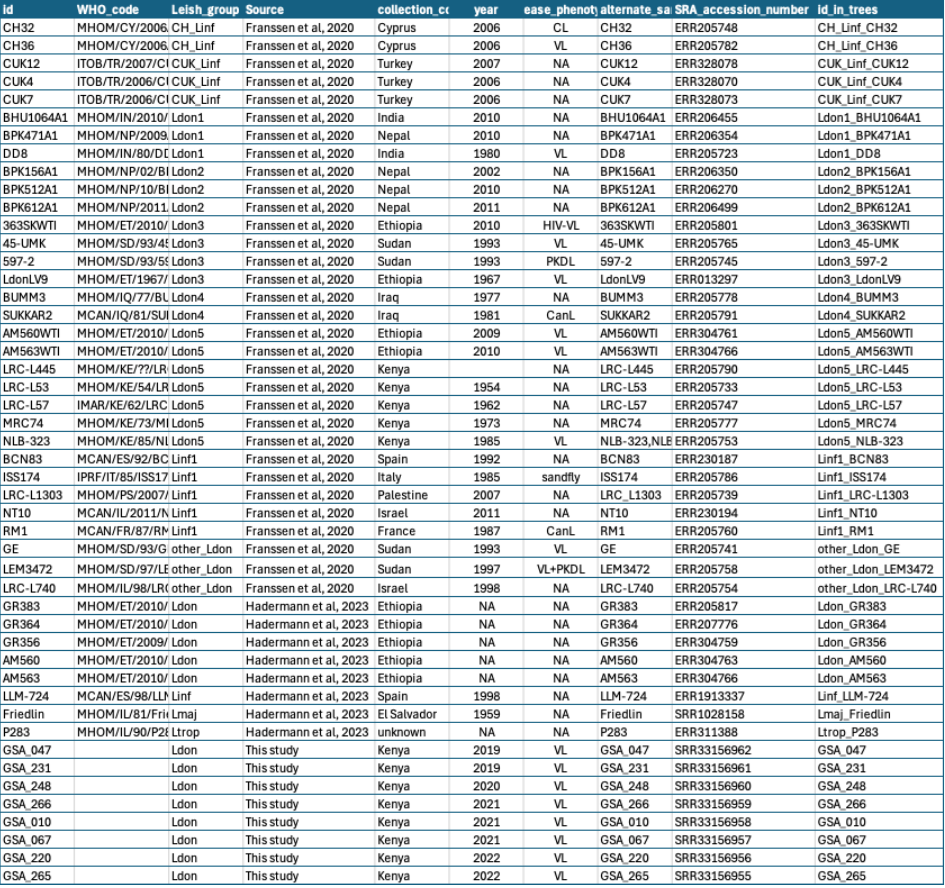

Supplement: S2 Table — (DOCX) [file pntd.0013144.s002.docx]

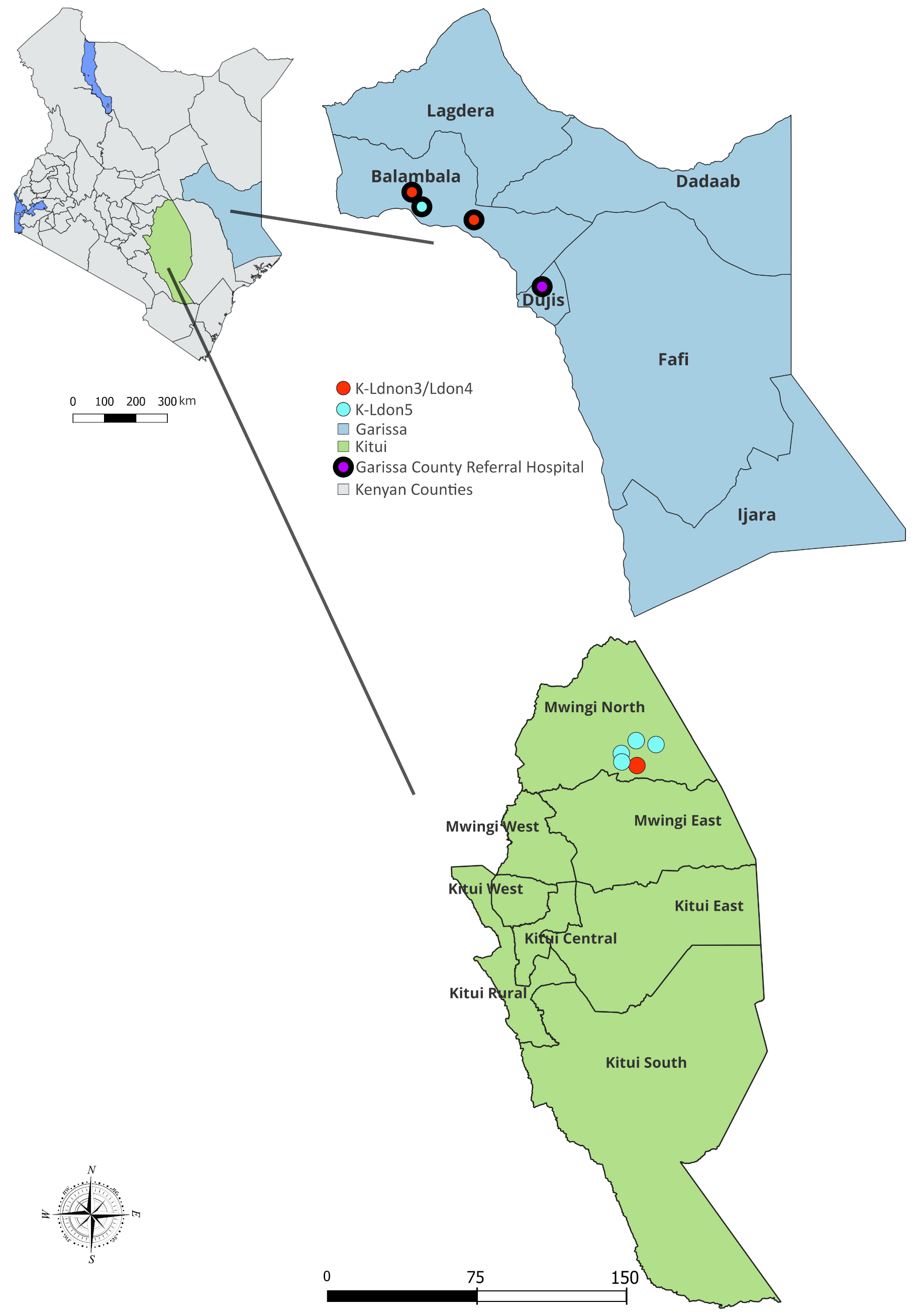

Supplement: S1 Fig — The dots indicate the reported origin of the 8 Leishmania samples (3 in Garissa and 5 in Kitui) analyzed by whole genome sequencing (WGS) by target enrichment (SuSL-seq). Samples clustered into two genomic groups: L. donovani group 5 (K-Ldon5: K for Kenya) similar to the previously reported L. donovani group 5. The other constituted a new genetic variant not reported previously (K- Ldon3/Ldon4). The map was generated using ArcGIS Pro 3.1. The base layer shapefile for Kenya’s administrative boundaries was obtained from openAFRICA (https://www.open.africa/dataset/kenya-counties-shapefile) and is licensed under the Creative Commons Attribution 4.0 International (CC BY 4.0) license). (TIF) [file pntd.0013144.s006.tif]

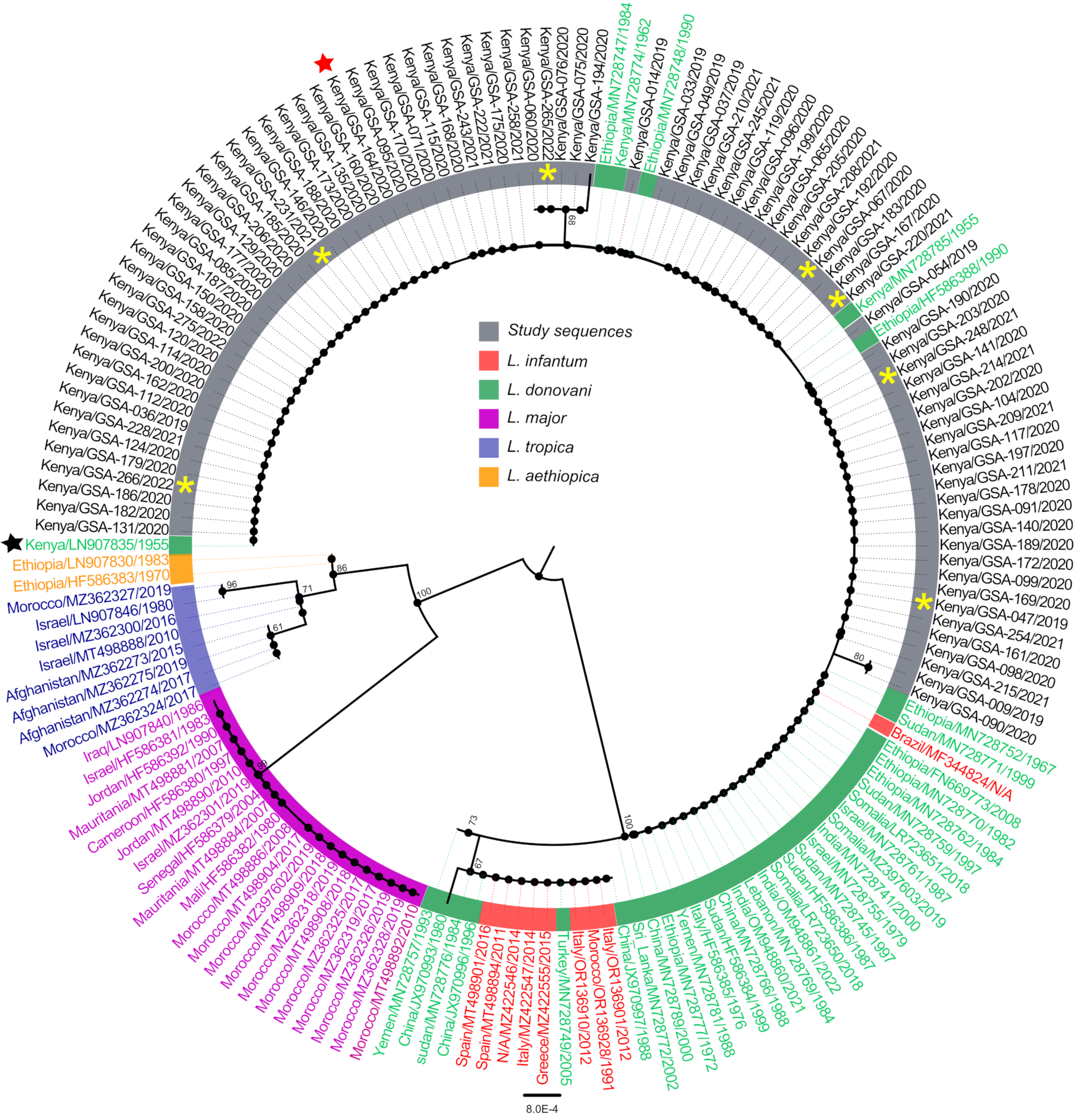

Supplement: S2 Fig — Black star shows a L. donovani sequence from Kenya that was deposited in GenBank in 1955 clustering with current study samples. The yellow stars shows 8 of the current study samples that were selected for targeted whole genome sequencing (Fig 2). Branch support was estimated using SH-like likelihood ratio test and are indicated as numbers. The scale bar represents the number of substitutions per site. (TIFF) [file pntd.0013144.s007.tiff]

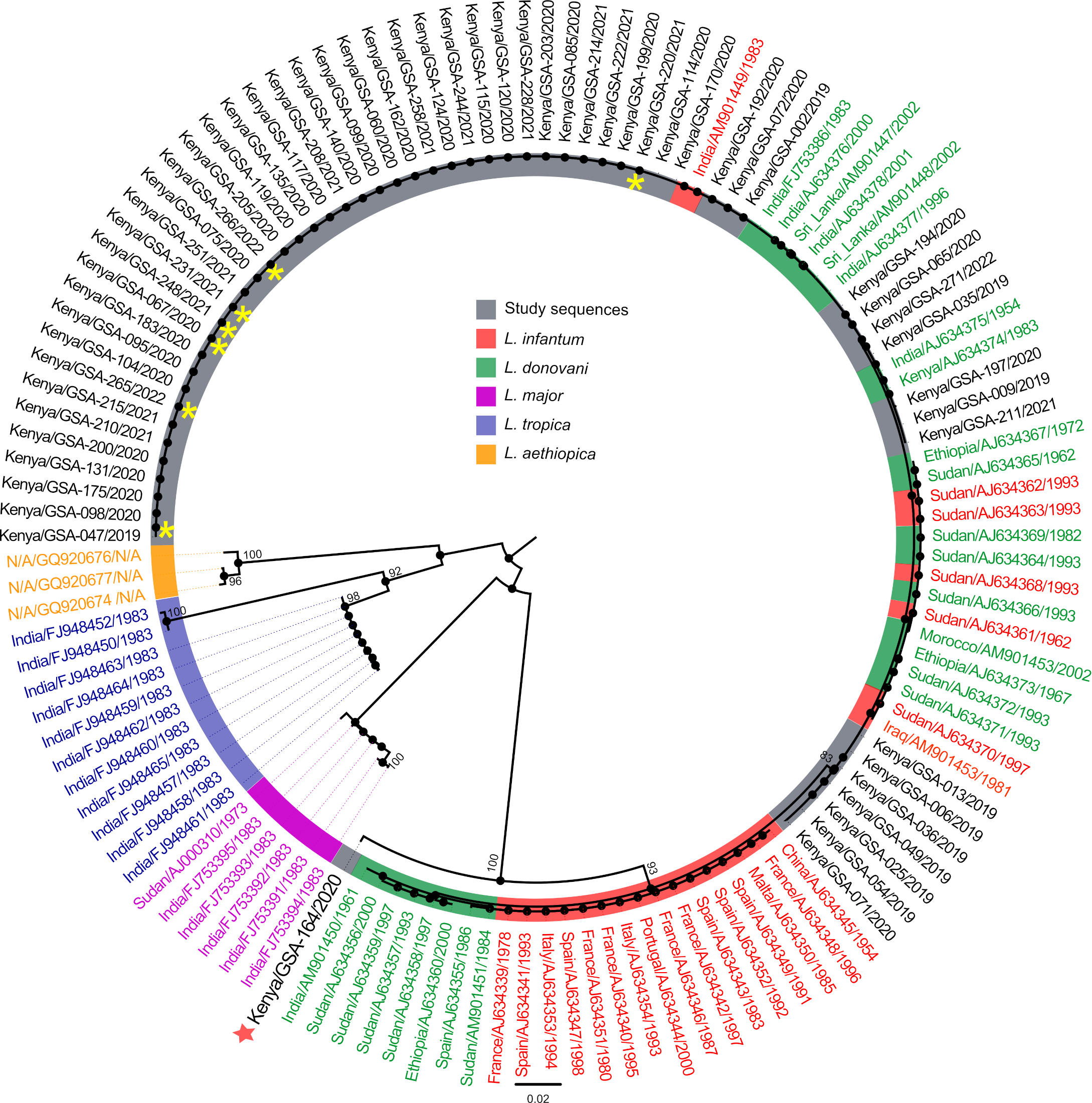

Supplement: S3 Fig — Red star shows an outlier study sample (Kenya/GSA-164/2020), separating distinctly from others. The yellow stars shows 8 of the current study samples that were selected for targeted whole genome sequencing (Fig 2). Branch support was estimated using SH-like likelihood ratio test and are indicated as numbers. The scale bar represents the number of substitutions per site. (TIF) [file pntd.0013144.s008.tif]

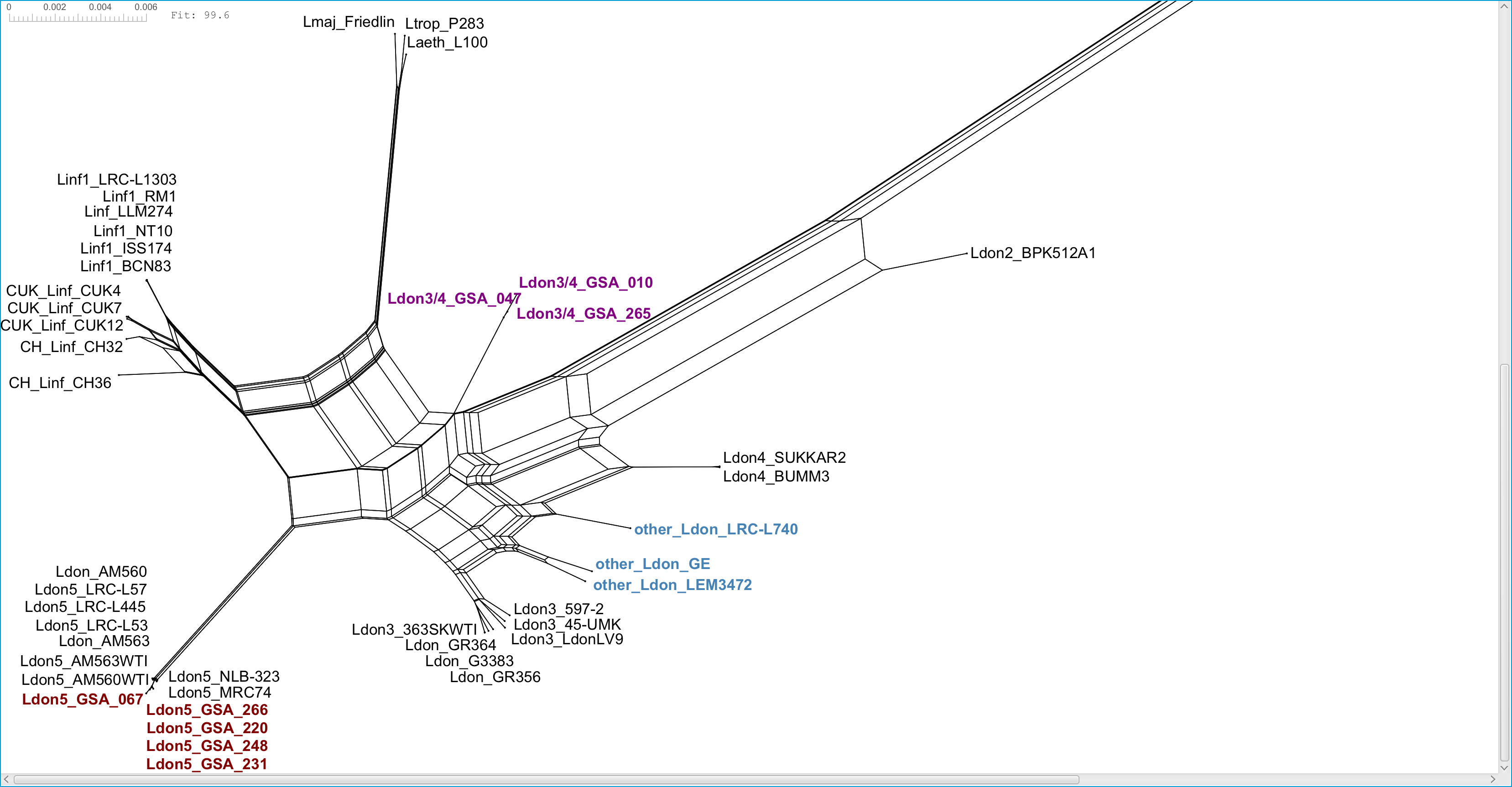

Supplement: S4 Fig — The samples in this study overlapping with cluster 5 L. donovani strains as defined by Franssen et al., [21] are shown in red. The three other strains not grouping with any of the known clusters are shown in a purple. (PNG) [file pntd.0013144.s009.png]

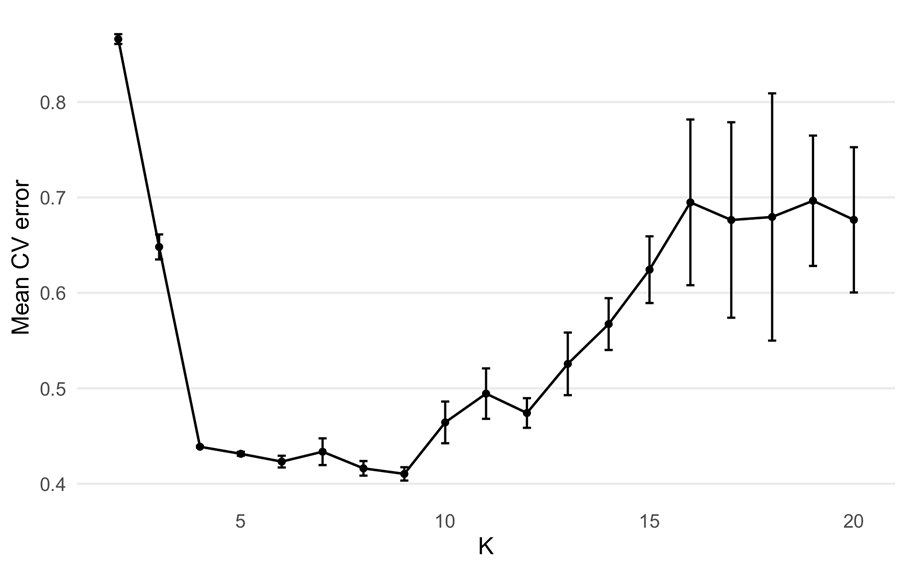

Supplement: S5 Fig — Cross validation error as extracted from the ADMIXTURE log files for each tested value of the number of populations (K). (PNG) [file pntd.0013144.s010.png]

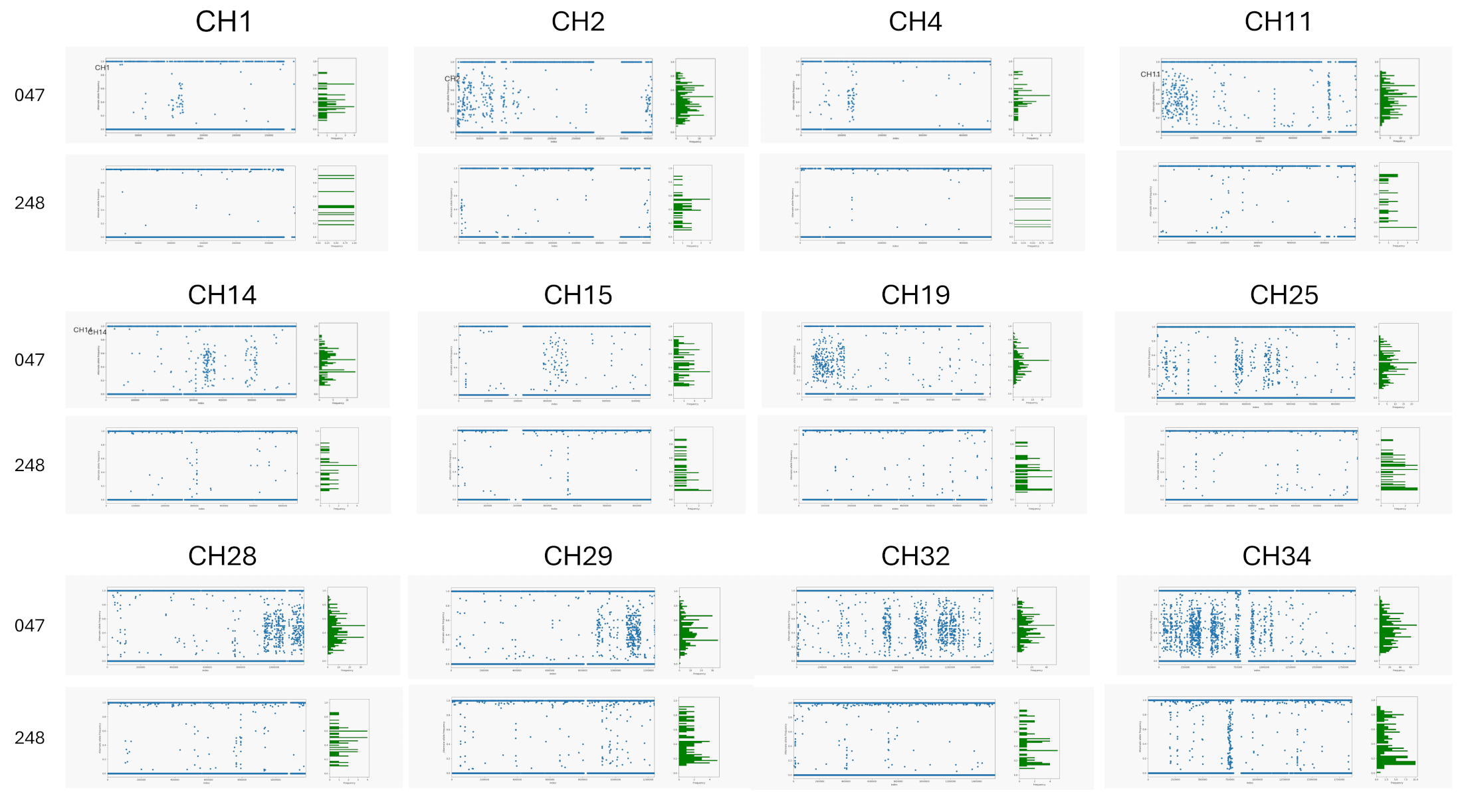

Supplement: S6 Fig — The X-axis represents the position of the SNP in the chromosome, and the Y-axis represents the allele frequency of the SNP in the corresponding strain. Presence of mixed ancestry signatures in a sample could be reflecting events of genetic exchange as well as polyclonality (mixture of different genotypes). To distinguish the two hypotheses, we plotted the alternative allele frequencies along the 36 chromosomes of admixed samples. In case of polyclonal mixture, alternative allele frequency should be constant all along the chromosomes and it should deviate from 50/50. While in case of genetic exchange, stretches of heterozygosity should be alternating with stretches of homozygosity (intra-chromosomal recombination) and in heterozygous stretches, the frequency should always be 50/50. The latter was observed for most chromosomes of admixed sample, not for the sample with single ancestry origin. Noteworthy, the analysis could only be done with samples with similar and high genome coverage (see S5 Table): samples GSA47 and GSA248 show a coverage > 10% in 65,5 and 85.6% of the genome respectively (the two other admixed samples GSA010 and GSA265 showed only 4.4 and 26.5% of genome coverage). (TIF) [file pntd.0013144.s011.tif]

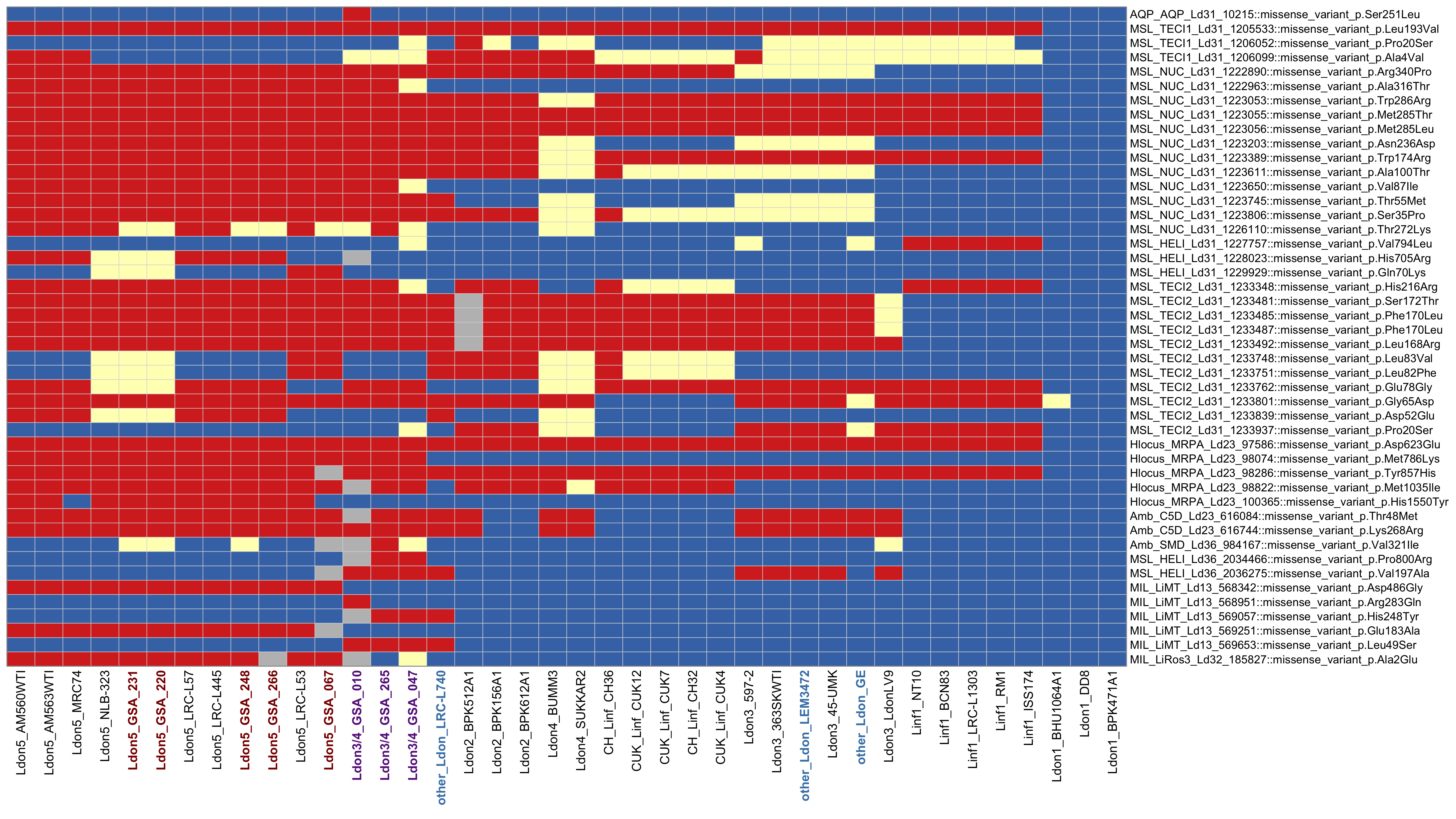

Supplement: S7 Fig — The color scheme represents different SNP categories: blue indicates the absence of SNPs, orange indicates heterozygous SNPs, and red indicates homozygous SNPs. The naming convention for SNPs follows the format of the gene of interest, position in the genome, type of mutation, and its effect on the corresponding protein. The study samples are shown in red and purple text. (PNG) [file pntd.0013144.s012.png]
