# Supplementary material for: Direct sequencing of Leishmania donovani from patients in Garissa County, Northern Kenya, reveals a newly emerging intra-specific hybrid genotype
Source: PLoS Negl Trop Dis. 2026 Jan 27;20(1):e0013144. doi: 10.1371/journal.pntd.0013144 (PMC12875589; doi:10.1371/journal.pntd.0013144)
Supplement: S3 Table — (DOCX) [file pntd.0013144.s003.docx]

| Period/Year | Total number of samples analyzed | Gender of patients with positive samples | |
| --- | --- | --- | --- |
|  |  | Male | Female |
| 2019 | 53 | 15 | 3 |
| 2020 | 153 | 64 | 21 |
| 2021 | 54 | 13 | 5 |
| 2022 | 26 | 6 | 1 |
| Total | 286 | 98 | 30 |
