# Supplementary material for: Direct sequencing of Leishmania donovani from patients in Garissa County, Northern Kenya, reveals a newly emerging intra-specific hybrid genotype
Source: PLoS Negl Trop Dis. 2026 Jan 27;20(1):e0013144. doi: 10.1371/journal.pntd.0013144 (PMC12875589; doi:10.1371/journal.pntd.0013144)
Supplement: Attachment — Submitted filename: Omwenga et al_Rebuttal_28Nov25.docx [file pntd.0013144.s014.docx]

PNTD-D-25-00754
Direct sequencing of *Leishmania donovani* from patients in Garissa County, Northern Kenya, reveals a newly emerging intra-specific hybrid genotype
PLOS Neglected Tropical Diseases

**Rebuttal Letter (text in bold corresponds to our answers to journal’s and reviewer’s comment**

PNTD-D-25-00754
Direct sequencing of Leishmania donovani from patients in Garissa County, Northern Kenya, reveals a newly emerging intra-specific hybrid genotype
PLOS Neglected Tropical Diseases

**Journal Requirements:**

1) Please provide an Author Summary. This should appear in your manuscript between the Abstract (if applicable) and the Introduction and should be 150-200 words long. The aim should be to make your findings accessible to a wide audience that includes both scientists and non-scientists. Sample summaries can be found on our website under Submission Guidelines:
<https://journals.plos.org/plosntds/s/submission-guidelines#loc-parts-of-a-submission>

**We have provided an author summary between lines 43-53 in the manuscript (MS) with tracked changes.**

2) Some material included in your submission may be copyrighted. According to PLOSu2019s copyright policy, authors who use figures or other material (e.g., graphics, clipart, maps) from another author or copyright holder must demonstrate or obtain permission to publish this material under the Creative Commons Attribution 4.0 International (CC BY 4.0) License used by PLOS journals. Please closely review the details of PLOSu2019s copyright requirements here: PLOS Licenses and Copyright. If you need to request permissions from a copyright holder, you may use PLOS's Copyright Content Permission form.
Please respond directly to this email and provide any known details concerning your material's license terms and permissions required for reuse, even if you have not yet obtained copyright permissions or are unsure of your material's copyright compatibility. Once you have responded and addressed all other outstanding technical requirements, you may resubmit your manuscript within Editorial Manager. 
Potential Copyright Issues:

- Figure S1. Please (a) provide a direct link to the base layer of the map (i.e., the country or region border shape) and ensure this is also included in the figure legend; and (b) provide a link to the terms of use / license information for the base layer image or shapefile. We cannot publish proprietary or copyrighted maps (e.g. Google Maps, Mapquest) and the terms of use for your map base layer must be compatible with our CC BY 4.0 license.
Note: if you created the map in a software program like R or ArcGIS, please locate and indicate the source of the basemap shapefile onto which data has been plotted.

**A detailed S1 Fig that has a direct link to the base layer of the map is now provided.**

If your map was obtained from a copyrighted source please amend the figure so that the base map used is from an openly available source. Alternatively, please provide explicit written permission from the copyright holder granting you the right to publish the material under our CC BY 4.0 license.
If you are unsure whether you can use a map or not, please do reach out and we will be able to help you. The following websites are good examples of where you can source open access or public domain maps:
* U.S. Geological Survey (USGS) - All maps are in the public domain. (<http://www.usgs.gov>)
* PlaniGlobe - All maps are published under a Creative Commons license so please cite u201cPlaniGlobe, <http://www.planiglobe.com>, CC BY 2.0u201d in the image credit after the caption. (<http://www.planiglobe.com/?lang=enl>)
* Natural Earth - All maps are public domain. (<http://www.naturalearthdata.com/about/terms-of-use/>).

**We have incorporated the source, link, and associated CC BY 4.0 for S1 Fig. We say “The map was generated using ArcGIS Pro 3.1. The base layer shapefile for Kenya’s administrative boundaries was obtained from openAFRICA (https://www.open.africa/dataset/kenya-counties-shapefile) and is licensed under the Creative Commons Attribution 4.0 International (CC BY 4.0) license.” See lines 629-640 in the Supporting Information.**

3) Please ensure that the funders and grant numbers match between the Financial Disclosure field and the Funding Information tab in your submission form. Note that the funders must be provided in the same order in both places as well.

**Done. See lines 443-449 in the MS with tracked changes.**

- State the initials, alongside each funding source, of each author to receive each grant. For example: "This work was supported by the National Institutes of Health (####### to AM; ###### to CJ) and the National Science Foundation (###### to AM).

**Done. See lines 443-449 in the MS with tracked changes.**

- State what role the funders took in the study. If the funders had no role in your study, please state: "The funders had no role in study design, data collection and analysis, decision to publish, or preparation of the manuscript.".

**Done**

If you did not receive any funding for this study, please simply state: u201cThe authors received no specific funding for this work.u201d.

**Not applicable**

**Reviewers' Comments:**

**R**eviewer #1: My first and most important concern is the ethical context of the work. Samples have been collected from Kenyan individuals. Yet, there has been no ethical review done and/or obtained from a Kenyan institute. An USA-based institute deemed that this was not needed, but I do not think that this is a leading opinion.

**We thank the reviewer for catching the missing details on ethical approvals. Our study was part of a public health surveillance activity, following a request by the Ministry of Health to provide diagnostic and laboratory support during a leishmaniasis outbreak in Garissa County. By definition, public health surveillance is not research and as such, consent was not obtained.** **The use of these routine surveillance data in research is considered acceptable because of the public health impact of research, the fact that risks to the subjects are minimized through data deidentification, and the impracticability of seeking retrospective consent (ref to the WHO guideline). The approval to provide diagnostic and laboratory support was approved by the Kenya Medical Research Institute Scientific and Ethics Review Unit (KEMRI SERU, # 4634) and the WRAIR Human Subject Protection Branch, (WRAIR #2699). We have revised the ethical statement to reflect these details. See lines 98-110 in the MS with tracked changes.**

Secondly for the WGS approach (8 samples) approval was obtained from ITM (Belgium), but this does not concern the use of the other specimen. I really think that an opinion from a Kenyan based institute should have been sought. This must be addressed by the authors.

**This important concern is now addressed in the revised manuscript. We say *“The approval to provide diagnostic and laboratory support, including WGS was approved by the Kenya Medical Research Institute Scientific and Ethics Review Unit (KEMRI SERU, # 4634) and the WRAIR Human Subject Protection Branch, (WRAIR #2699)”.* See lines 103-106 in the MS with tracked changes. ITM issued a separate approval for the samples sequenced at ITM.**

The authors should also explain why only 8 samples were selected for WGS. Is this number sufficient to support the conclusions. The rationale should be presented in M&M section, its impact on the conclusions in the discussion.

**The WGS arm of the study was a proof-of-principle aiming to demonstrate the applicability of our protocol of direct genome sequencing in clinical samples (here blood) of patients with VL. We clearly showed that samples with high parasitemia could successfully be sequenced. The genome diversity we already observed in the small sample analyzed in this pilot study justifies further studies on larger number of samples. This information has now been included in different places of the revised MS.**

- **Line 38: “*Conclusion: This pilot study reveals*…”**
- **Line 150: “*In this proof-of-principle study, eight samples with high parasitemia*…”**
- **Lines 278-279 “*Based on parasite load, eight blood samples with Ct values of <33 were selected (S5 Table) for attempting direct targeted sequencing by SuSL-seq. Genome sequencing was successful for all of them, with percentage of genome coverage > 5x ranging between 21.30 % and 88.39 % (average 67.97 %)*”.**
- **Lines 379-380: “*We also combined target enrichment and WGS (SuSL-seq) for a pilot exploration of the Leishmania genome in 8 of the study samples*”**
- **Lines 419-433: “*Our pilot study sheds some light on the genetic diversity within L. donovani complex, especially in East Africa where VL has become a hot spot. The observation……… In addition***
- **Lines 427-431: *“Further research on a larger sample size is needed to fully document the genomic heterogeneity of L. donovani in Kenya in past and current outbreaks (like Wajir and Pokot). Extension is also needed towards surrounding countries, in order to assess among others the epidemiological dynamics and the dispersal/spread of parasites across the continent and between continents (for instance Africa and the Indian sub-continent)”.***

Reviewer #2: Study design, methods, statistical analysis is correctly used. For ethics, in this study, it is stated that the work was conducted as part of the public health response during the outbreak. I am wondering, in addition to the routine diagnosis, whether research use of the samples, such as WGS is also included in this permission? If the Kenyan ethical review committee has determined that such approval is not necessary, it would be advisable to clearly state this to avoid any potential misunderstandings.

**Reviewer 1 had the same concern. This important concern is now addressed in the revised manuscript. See lines 98-110 in the MS with tracked changes.**

Reviewer #3: Methods presented are ok, but some missing key experimental desing are needed in the current state

**Results**


-Does the analysis presented match the analysis plan?

-Are the results clearly and completely presented?

-Are the figures (Tables, Images) of sufficient quality for clarity?

Following the review, we have improved the quality and clarity of Figures and Tables.

Reviewer #1: Table 1 is not very informative, possibly it could be removed. If maintained more demographic information must be added. Also the heading should specify that it concerns a specific locality.

**We agree with the reviewer. We moved the Table to supplementary information (See S3 Table). The information is also captured in the text. See lines 246-251 in the MS with tracked changes.**

Reviewer #2: Results align withs the methods and analysis plan, results are clearly and completely presented. The figures are well-designed and thoughtfully constructed, which helps to simplify the interpretation of the results. This effectively conveys the clarity of the findings.

**Thanks for this comment**

Reviewer #3: Can be improved

**The new version of the MS has better alignment between the methods, analysis plan and results. In the section titled “Study Site and Design, we have added a sentence that says “This study applied WGS to characterize *Leishmania* directly from patient blood samples, eliminating the need for parasite isolation and culture”. See lines 114-116 in the MS with tracked changes.**

**Conclusions**


-Are the conclusions supported by the data presented?

-Are the limitations of analysis clearly described?

-Do the authors discuss how these data can be helpful to advance our understanding of the topic under study?

-Is public health relevance addressed?

Reviewer #1: Samples have been collected about 5 years ago and from a specific region. Nowadays Kenya is confronted with other major outbreaks, Wajir, Pokot. How do the authors think that the results of their work translate to these recent outbreaks?

**The reviewer raises a very pertinent question that should be thoroughly explored and our paper shows that this is possible at an unprecedented resolution with our sequencing approach.**

- **In multiple places we say how our findings could be expanded to inform other regions in Kenya with similar outbreaks.**
- **Lines 405-406 we have added a sentence that says: “Accordingly, analysis of additional samples is required to consider (re-)naming Ldon3/Ldon4 and in the meantime, it is more informative to keep the naming based on the combination of defined ancestry patterns”.**
- **Lines 427-431 we say: “****Further research on a larger sample size is needed to fully document the genomic heterogeneity of *L. donovani* in Kenya in past and current outbreaks (like Wajir and Pokot). Extension is also needed towards surrounding countries, in order to assess among others the epidemiological dynamics and the dispersal/spread of parasites across the continent and between continents (for instance Africa and the Indian sub-continent).”**

Reviewer #2: See general comment

Reviewer #3: Needs revision

**We have revised the paper extensively**

Use this section for editorial suggestions as well as relatively minor modifications of existing data that would enhance clarity. If the only modifications needed are minor and/or editorial, you may wish to recommend “Minor Revision” or “Accept”.

Reviewer #1: See my comment above on ethics. This must be addressed

**This important comment was addressed. See lines 98-110 in the MS with tracked changes.**

Reviewer #2: Line 305
"For, instance" should be corrected to "For instance".

**Done. See line 362 in the MS with tracked changes.**

Figure 3 – Color and Labeling
The yellow area missing strain names. Also, the orange representing heterozygous SNPs seems to be yellow, which could be confusing.

**We think the reviewer is referring to Fig 4. This figure is now S7 Fig). The missing strain names have been added.**

Supplementary Table S3
It would be helpful to include the percentage of parasite reads for each sample. Including Ct values as well may allow readers to assess whether enrichment efficiency correlates with the initial parasite load or is influenced by other factors such as sample DNA quality.

**Done. This Supplementary Table is now S5 Table. The qPCR Ct values have been added.**

Figure 3
Consider marking GSA-164 with a red star to improve readability.

**This figure is now S3 Fig. “GSA-164” is marked with a red star. We also show “GSA-164” in S2 Fig.**

Reviewer #3: (No Response)

**Summary and General Comments**

Use this section to provide overall comments, discuss strengths/weaknesses of the study, novelty, significance, general execution and scholarship. You may also include additional comments for the author, including concerns about dual publication, research ethics, or publication ethics. If requesting major revision, please articulate the new experiments that are needed.

Reviewer #1: This is a well written and presented paper. It provides a significant insight in the genetic diversity of *Leishmania* parasites relevant for Kenya, but it is of interest beyond this region.

Reviewer #2: The authors conducted a detailed genetic analysis of Leishmania donovani outbreak strains in Kenya using a target enrichment approach. This method enabled high-resolution phylogenetic analysis that would not have been possible through conventional single gene sequence analysis. Their results reveal that the outbreak did not originate from a single clonal lineage but instead consisted of genetically at least two distinct parasite populations. Furthermore, these populations appear to constitute a novel group that does not cluster with any previously known groups, and the authors propose to designate this as a new clade, “Ld6”. Importantly, genome-wide SNPs obtained directly from clinical samples also suggest potential drug resistance. These findings provide significant implications for treatment strategies and future outbreak responses in Kenya. The study is well-conducted, and the manuscript is clearly written and easy to follow.

Line 222: GSA-164
The authors discuss GSA-164, which showed divergent behavior in ITS and HSP-based phylogenetic analyses, possibly due to low Ct values. While HSP sequences showed no substantial difference from other Ld strains in this study, ITS sequences appear to be highly divergent. The phylogenetic distance observed in the ITS sequence is unusually large, making interpretation difficult. According to the methods, the ITS amplicon was sequenced using Illumina MiSeq following library preparation, which likely included enzymatic fragmentation and subsequent clustering by bioinformatics processing. The authors should briefly describe how the consensus sequence for this sample was generated. Was a single, unambiguous ITS consensus sequence obtained for this sample? Could there be a minor component in the original sample—such as L. major or another heterologous population—that introduced potential chimeric sequences either through PCR template switching or during downstream analysis?

**We agree with reviewer that in general, the ITS sequences are more divergent than those derived from Hsp70. We offered an explanation for this discrepancy in the discussion. We said that Hsp70 is a conserved housekeeping gene while the ITS is extensively variable (See lines 350-358).**

**The reviewer is correct on how the ITS and indeed the Hsp70 sequences were generated and therefore the possibility of chimera cannot be ignored. In the methods section, we have elaborated on the method used to check for chimera. We say “*Taxa with unusually long branches had their consensus sequences re-evaluated for chimeras using UCHIME v4.2.40 against the QIIME eukaryotic database (release 2025-02-19), and the non-chimeric reads used for consensus generation”.* See lines 197-203, 265-270 and 375-378 in the manuscript with tracked changes.**

Ct Threshold
The authors used a Ct cutoff of 33 for sample selection for SureSelect, with successful results. Is there a rationale or empirical basis for selecting this Ct threshold? A brief explanation should also be provided regarding why whole-genome sequencing could not be performed for GSA-164.

**The genus specific qPCR (with Ct values ranging between 26 and 33) was used to screen and detect samples with high parasite load. For SuSL-seq, two further criteria were added: the total DNA concentration and the % of *Leishmania* DNA. This is now clarified in lines 150-156 in the manuscript with tracked changes.**

**We say *“In this proof-of-principle study, eight samples with high parasitemia as measured by genus-specific PCR (Ct values between 26-33) were selected for WGS. For SuSL-seq, a minimum of 10 ng of total DNA and 0.006% of Leishmania DNA are required as described by*** [23]***. Total DNA was measured by qubit fluorometer (Thermo Fisher Scientific, USA). We used 21-50ng of total DNA for library preparation. Leishmania DNA quantification was then performed by qPCR as described by*** [23]: ***Leishmania DNA in the samples ranged from* 0.0064 to 0.1415 *%”.***

Discussion
The authors might consider elaborating on the ecological background that could explain the occurrence of hybridization within the Ld lineage. Assuming that L. donovani is anthroponotic with no animal reservoir, the opportunities for recombination in nature would be quite limited, likely requiring co-infection of sandflies that fed on multiple human hosts. This would imply that such events are rare and not frequently occurring.

**Actually, several recent studies based on WGS show that hybrids are not so rare in natural populations; see for instance in *L. braziliensis* in Peru** [60]**, *L. aethiopica* in Ethiopia** [43]***, L. tropica* in Morocco** [42]***,* and *L. donovani* in Nepal [in preparation]. This could be explained by fact that more studies are being completed on sympatric samples and by the high discriminatory power of WGS. With respect to the opportunities for recombination in nature, co-infection of sand flies that fed on multiple human hosts is indeed likely to be exceptional, but we would like to remind that polyclonal infections in human hosts is rather common ( 9% in primary VL isolates and 14% in recurrent VL, and this could lead to polyclonal infections in the sand fly and a potential source of hybrids. This is briefly discussed in the revised paper, see lines 413-418 in the manuscript with tracked changes.**

**“In the past, sexual recombination in *Leishmania* was thought to be rare, but this topic was highly debated** [59]**. Recent studies show that hybrids are not that rare in natural populations; see for instance in *L. braziliensis* in Peru** [60]**, L. aethiopica in Ethiopia** [43]**, *L. tropica* in Morocco** [42]**, *L. donovani* in India** [63] **and *L. infantum/L. donovani* in Italy** [62]**. We believe that this is due (i) to sampling which is more sympatric than before and (ii) to the highest discriminatory power of WGS.”**

Reviewer #3: This is an interesting and timely epidemiological/genomic study. To my knowledge, it is the first application in Africa of targeted enrichment directly from patient blood to sequence Leishmania genomes, avoiding culture bias and enabling insights into population structure and potential drug-resistance signatures. These contributions are important for public health surveillance. I have several substantive comments that, if addressed, will strengthen the paper’s impact and technical clarity.

Major comments

- Frame the study around the Ld1–Ld5 (Franssen) subdivision and why it matters
The Introduction nicely summarizes VL epidemiology, but it doesn’t prepare the reader for the paper’s central population-genetic result which is placement of Kenyan isolates relative to the Ld group framework and the proposal of an additional “Ld6-like” set (Results/Discussion, including Fig. 2 and related text). Please expand the Introduction to (i) explain why Ld groupings (Franssen et al.) are a useful organizing principle; (ii) set expectations that multi-locus or whole-genome SNP phylogenies integrate signals across many genes (more pangenome-like than single-marker trees), and (iii) preview why a putative new cluster would be meaningful beyond relabeling. This context will make later sections more interpretable.

**Indeed, this would facilitate the interpretations of later sections. We added a paragraph in the introduction. See lines See lines 80-85 in the manuscript with tracked changes.**

**“In 2020, a phylogenetic classification of the two species of the donovani complex, based on WGS analysis of 151 cultured isolates, was published** [21]**. Accordingly, 5 groups were identified in *L. donovani* (Ldon1-5), 3 of them being endemic in Africa and Middle East and the two others in the Indian sub-continent. WGS offers a pan-genome perspective on parasite evolution with a high discriminatory power. This offers a unique reference frame for several applications like tracking the source of outbreaks, monitoring transmission patterns or identifying new genotypes.”**

**We also re-contextualized the subsequent paragraph as suggested by the review. See lines 86-90.**

**“Present study used targeted gene sequencing and untargeted WGS to directly characterize the Leishmania parasites collected from patients’ blood at Garissa County Referral Hospital, in Northeastern Kenya during the 2019 - 2022 VL outbreak and situate them in the phylogenetic reference frame mentioned above. Noteworthy, we applied here for the first time in Africa, a method for direct genome sequencing of *L. donovani* in blood samples from VL patient, without the need for parasite isolation and culture** [23,24]**.”**

- Methods for targeted enrichment: describe probe design and report capture performance
The Methods specify a custom Agilent SureSelect design (SuSL design ID S3377046; ~318k 120-mer probes covering ~29.99 Mbp) and NovaSeq 2×150 bp sequencing, which is excellent. Please clarify whether this probe set is newly designed here or previously published and, in either case, cite the design source and criteria (tiling density, target regions, species coverage). Beyond read counts, the manuscript should report standard capture metrics per sample: on-target rate, mean depth, duplication rate (post-dedup), and breadth of coverage. Provide per-chromosome (or genome-wide) coverage plots and relate capture success to Ct values (you selected Ct 26–33; Methods). This QC is essential for judging how robustly SuSL-seq worked on blood and how that impacted downstream analyses (e.g., allele-frequency plots rely on adequate coverage).

**Details on the SureSelect design are now included in the text, see lines 161-168. We say:**

***“The probe design used in this study was based on a previously published SureSelect design. Briefly, to generate the custom Agilent SureSelect (SuSL) capture array, repetitive and low-complexity regions of the reference genome (Leishmania donovani BPK282A1) were masked prior to probe selection. Candidate 120-mer probes were designed at 1x tiling density to minimize overlap, avoiding regions with ambiguous bases (Ns) and excessive sequence redundancy. To ensure high specificity, all probes were aligned using BLAST against the genome of Homo sapiens; any probes showing significant homology to these genomes were excluded to prevent off-target hybridization. The final design (Agilent SuSL design ID S3377046) comprised approximately 318,000 probes, collectively targeting ~29.99 Mb of the reference genome”.***

**Standard capture and sequencing metrics were already included in the original manuscript (S5 Table). For each sample, we report the total number of raw reads, mapped reads, and high-quality reads retained after filtering for mapping quality (mapQ > 30) and removal of duplicates. We also provide the percentage of the genome covered at >5x, >10x, and >25x, as well as the median and average sequencing depth. These metrics collectively describe the on-target rate, duplication rate (post-deduplication), and breadth and depth of coverage across all samples. The qPCR Ct values selected (Ct 26–33) have been incorporated in S5 Table.**

- Case definition and consistency (“suspected” vs. “confirmed”)
The Abstract and some Methods describe samples from “patients suspected of having VL,” yet “Study site and design” states “patients with visceral leishmaniasis” while only 128/286 (45%) were Leishmania-positive by AAP3 qPCR (Results, Table 1). Please standardize to “patients suspected of VL” in the body text for accuracy and consistency with your own numbers.

**This has been corrected in the method section under “Study site and design” line 112**

- Amplicon marker success and rationale
You recover 86 Hsp70 and 79 ITS sequences (Results). Please comment on why yields differ (e.g., DNA quality, Ct, primer mismatch) and provide a simple success-rate summary by Ct strata. A small supplemental table or figure relating Ct to amplicon success would make the targeted marker section more informative. In summary: Add a supplemental CT table for all samples (amplicon and WGS-selected), plus a coverage summary table for the eight WGS samples: raw reads, % on-target, mean depth, % genome, % duplicates. Include a coverage-vs-Ct scatterplot to illustrate performance boundaries of SuSL-seq in blood.

**The reason for differential success rate between the Hsp70 and ITS is now provided in lines 350-358. We say:**

**In the current study, *Leishmania* genomes were first analyzed by targeting the Hsp70 and ITS loci. We observed a slightly higher amplification success for Hsp70 (n=86) compared to ITS (n=79), which we attribute to the intrinsic molecular properties of the targets and their respective primers. Hsp70 is a conserved housekeeping gene that is essential for the parasite's survival and adaptation to various environmental stresses and has been widely used for phylogeny and taxonomy of Leishmania parasites** [53,54]**. It is highly conserved and thus has less chance of primer mismatches to target region. ITS region is extensively variable and is widely used for identification as well as phylogenetic analysis of *Leishmania* spp.** [55]. **Its high variability leads to better species discrimination, but at a cost of higher chance of primer mismatches that can reduce amplification yield.**

**We have added S4 Table to show the relationship between *Leishmania* spp Ct values and amplicon success for both *Hsp70* and ITS markers. S5 Table has been updated to include coverage statistics for the eight WGS samples (raw reads, % on-target, mean depth, % genome covered, and % duplicates). Additionally, a coverage versus Ct scatterplot has been incorporated within the same supplementary file (S5 Table) to illustrate the performance boundaries of SuSL-seq in blood samples.**

- Population structure and the “new cluster”
Your ADMIXTURE and phylogenomic analyses suggest three Kenyan samples sit between Ld3 (Sudan/Ethiopia) and Ld4 (Iraq), with admixture like previously noted hybrids. Given the small WGS sample size and the authors’ own acknowledgement that the group is not monophyletic (Discussion), I recommend softening the claim from “new cluster Ld6” to “an admixed genotype” (or similar) unless additional sampling or tests are added to support a stable, reproducible group. The Discussion should also note that recombination and polyclonality can both produce mixed ancestry patterns; your allele-frequency analysis is a good step build on it with clearer thresholds/criteria.

**We fully agree and have toned down the claim. See lines 398-406. Importantly, we have added a concluding statement as follows (lines 405-406): *“Accordingly, analysis of additional samples is required to consider (re-)naming Ldon3/Ldon4 and in the meantime, it is more informative to keep the naming based on the combination of defined ancestry patterns.”***

**In lines 407-411, we have expanded the discussion on the admixture. We say: “*The observation of mixed ancestry patterns could reflect recombination or polyclonality. The analysis of alternative allele frequencies along the 36 chromosomes of admixed samples allowed distinguishing the two hypotheses. In case of hybridization, stretches of heterozygosity should be alternating with stretches of homozygosity (intra-chromosomal recombination) and in heterozygous stretches, the frequency should always be 50/50*** [58]***. In case of polyclonal mixture, there should be no alternation of homozygous/heterozygous stretches and alternative allele frequency could deviate from 50/50.***

-Drug-resistance SNPs: present as genomic signals unless functionally validated
The heatmap of SNPs in 11 AMR-implicated genes is eye-catching, but without phenotypic or epidemiological correlation it should be framed as putative genomic signatures. If phenotypic validation is not feasible now, consider moving the heatmap and gene-by-gene calls to the Supplement and dialing back claims in the main text, or add stronger caveats in Results/Discussion.

**We concur with reviewer’s sentiments. Section of the results on drug resistance together with Fig.4, now S7 Fig were moved to Supplemental information. The discussion was also modified to include the sentiments of the reviewer. See lines 422-427. We say:**

***“In addition, in the selected genes reported to be involved in resistance to Antimony, Amphotericin B and Miltefosine*** [46-50]***, we identified a total of 46 SNPS in all the 8 Kenyan samples. Nevertheless, the presence of these drug resistance genes should be interpreted with care: at this stage, the genes represent genomic signatures and further phenotyping work is required to understand their functional implication in resistance.”***

- Show the link between LD pruning, SNP counts, and ADMIXTURE model choice
You prune with PLINK and evaluate K=4–11 by CV error. Please (i) justify the LD threshold (r²=0.5 is fairly permissive), (ii) report the final SNP count after pruning, and (iii) include the CV error values with error bars or replicate runs to show stability. This will help readers evaluate the K choice and ancestry patterns.

**Thanks for these suggestions. We have adjusted the statements accordingly. see lines 230-235.**

**“For downstream population genomic analysis, SNP pruning for linkage disequilibrium (LD) pruning was performed using PLINK2 (--indep-pairwise 50 10 0.5) with a threshold of r² = 0.5, which was selected to retain sufficient SNPs for population structure inference while minimizing the impact of tightly linked variants. This relatively permissive threshold was chosen because of the moderate LD decay observed in previous Leishmania genomic epidemiology studies** [42-44] **and to maintain representation across the genome despite local variation in recombination rates.”**

**See also lines 308-314**

***“After LD pruning, a total of 4050 SNPs remained and were used as input for ADMIXTURE analysis. We evaluated K = 2–20 using fivefold cross-validation. ADMIXTURE version 1.3.0 computes point estimates only, without standard errors, as noted in the log output. To assess model stability, we repeated each K value across 10 independent runs with different random seeds and obtained consistent ancestry proportions among replicates. The mean CV error values for each K are now visualized including the standard error in S5 Fig, showing that CV error reached a minimum with K-values ranging between 4 and 9.”***

Minor comments

Line ~93 (Study site/design): Replace “patients with visceral leishmaniasis” with “patients suspected of visceral leishmaniasis,” consistent with your Abstract and 45% qPCR positivity.

**Done.**

Lines ~103–105 (AAP3 qPCR mix): “Meridian Bioscience, USA” is duplicated—please remove the duplicate and keep the one with web link once.

**Done. See line 125**

Line ~125 (MiSeq): Specify “MiSeq v3 600-cycle kit (2×300 bp)” explicitly to be consistent with the 2×150 bp NovaSeq run later (line ~141).

**Thanks for catching this omission. The statement now reads “using v3 600 cycle kit; 2×300 bp paired reads. See lines 147-148 in the MS with tracked changes.**

Line ~154 (MAFFT): Add the MAFFT version (and algorithm, e.g., L-INS-i/G-INS-i) used via the Geneious plugin.

**Done. See lines 187-188.**

Lines ~172–183 (Mapping & GATK): You list GATK filters but no minimum depth; please state a DP threshold (e.g., DP ≥10) used in filtering and in downstream analyses.

**In our variant filtering, we did not apply a direct depth (DP) threshold, as the GATK filter “QD < 2.0” (Quality by Depth) already normalizes variant quality by the depth of coverage. This approach effectively accounts for low-depth sites without imposing a strict depth cutoff, ensuring that only variants with sufficient read support relative to their quality are retained.**

Line ~181: Add SnpEff version

**Done. See line 220**

Line ~186: Add RAxML/RAxML-NG version.

**Done. See lines 225**

Line ~189: Add SplitsTree4 version.

**Done. See lines 228**

Line ~192: Add ADMIXTURE version and random seed(s) for replication.

**Done. See lines 235**

Line ~198: Add BCFtools version.

**Done. See lines 241**

Figures: Improve resolution/legibility and enforce consistent nomenclature. For example, use a single convention (e.g., Ld5) across text and figures instead of mixing Ld5/Ldon5. Also ensure sample labels match between Fig. 2 and Fig. 3 and that axis labels and legends are readable at journal scale.

**Done**

PLOS authors have the option to publish the peer review history of their article ([what does this mean?](https://track.editorialmanager.com/CL0/https:%2F%2Fjournals.plos.org%2Fplosntds%2Fs%2Feditorial-and-peer-review-process%23loc-peer-review-history/1/010f01997d6f5e9d-d13d96df-ec68-46be-a026-d8c34ff04ad7-000000/_o9euYmLvJDUiCrAACaDDJKePV84CUYUwKV2NOLoyFA=230)). If published, this will include your full peer review and any attached files.


If you choose “no”, your identity will remain anonymous but your review may still be made public.


**Do you want your identity to be public for this peer review?** For information about this choice, including consent withdrawal, please see our [Privacy Policy](https://track.editorialmanager.com/CL0/https:%2F%2Fwww.plos.org%2Fprivacy-policy/1/010f01997d6f5e9d-d13d96df-ec68-46be-a026-d8c34ff04ad7-000000/sQTsxkBF_PNB1JK124O0lXhb-HRMCq1PVb7MPU8kCss=230).

Reviewer #1: No

Reviewer #2: No

Reviewer #3: No

[NOTE: If reviewer comments were submitted as an attachment file, they will be attached to this email and accessible via the submission site. Please log into your account, locate the manuscript record, and check for the action link "View Attachments". If this link does not appear, there are no attachment files.]

**Figure resubmission:**

 While revising your submission, we strongly recommend that you use PLOS’s NAAS tool (<https://ngplosjournals.pagemajik.ai/artanalysis>) to test your figure files. NAAS can convert your figure files to the TIFF file type and meet basic requirements (such as print size, resolution), or provide you with a report on issues that do not meet our requirements and that NAAS cannot fix.

After uploading your figures to PLOS’s NAAS tool - <https://ngplosjournals.pagemajik.ai/artanalysis>, NAAS will process the files provided and display the results in the "Uploaded Files" section of the page as the processing is complete. If the uploaded figures meet our requirements (or NAAS is able to fix the files to meet our requirements), the figure will be marked as "fixed" above. If NAAS is unable to fix the files, a red "failed" label will appear above. When NAAS has confirmed that the figure files meet our requirements, please download the file via the download option, and include these NAAS processed figure files when submitting your revised manuscript.

**Reproducibility:**

To enhance the reproducibility of your results, we recommend that authors of applicable studies deposit laboratory protocols in protocols.io, where a protocol can be assigned its own identifier (DOI) such that it can be cited independently in the future. Additionally, PLOS ONE offers an option to publish peer-reviewed clinical study protocols. Read more information on sharing protocols at <https://plos.org/protocols?utm_medium=editorial-email&utm_source=authorletters&utm_campaign=protocols>
